# External validation of the accuracy of cardiovascular risk prediction tools in psoriatic disease: a UK Biobank study

**DOI:** 10.1007/s10067-025-07325-y

**Published:** 2025-01-20

**Authors:** David M. Hughes, Zenas Z. N. Yiu, Sizheng Steven Zhao

**Affiliations:** 1 Department of Health Data Science, University of Liverpool, Liverpool, UK; 2 Centre for Dermatology Research, Northern Care Alliance NHS Foundation Trust, The University of Manchester, Manchester Academic Health Science Centre, National Institute for Health and Care Research Manchester Biomedical Research Centre, Manchester, UK; 3 Centre for Musculoskeletal Research, Division of Musculoskeletal and Dermatological Science, School of Biological Sciences, Faculty of Biological Medicine and Health, The University of Manchester, Manchester Academic Health Science Centre, Manchester, UK

**Keywords:** Cardiovascular risk prediction, Psoriasis, Psoriatic arthritis, Rheumatoid arthritis, UK Biobank, Validation

## Abstract

**Introduction:**

Risk prediction is important for preventing and managing cardiovascular disease (CVD). CVD risk prediction tools designed for the general population may be inaccurate in people with inflammatory diseases.

**Objectives:**

To investigate the performance of four cardiovascular risk prediction tools (QRISK3, Framingham Risk Score, Reynolds Risk Score and SCORE) in psoriatic arthritis (PsA) and psoriasis. We also compare performance in participants with no inflammatory conditions and in people with rheumatoid arthritis (RA).

**Methods:**

This research utilised the UK Biobank Resource. We identified participants with PsA, psoriasis and RA and calculated their cardiovascular risk using each risk tool. We assessed model calibration by comparing observed and predicted outcomes. Discrimination of 10-year risk prediction was assessed using time-dependent area under ROC curve (AUC), sensitivity, specificity, positive and negative predictive values.

**Results:**

We included 769 individuals with PsA, 8062 with psoriasis and 4772 with RA when assessing the QRISK3 tool. Predictions for individuals with psoriasis were roughly as accurate as those with no inflammatory conditions with time-dependent AUC of 0.74 (95%CI, 0.72, 0.76) and of 0.74 (95%CI, 0.72, 0.77) respectively. In contrast, individuals with PsA obtained the least accurate predictions with an AUC of 0.70 (95%CI, 0.64, 0.76). Individuals with RA also obtained less accurate predictions with AUC of 0.72 (0.69,0.74). For the Framingham risk score, AUCs varied between 0.61 (95%CI, 0.55, 0.68) for participants with PsA and 0.71 (95%CI, 0.68, 0.74) for individuals with no inflammatory condition.

**Conclusions:**

In general, CVD risk prediction accuracy was similar for individuals with psoriasis or no inflammatory condition, but lower for individuals with PsA or RA.

**Supplementary Information:**

The online version contains supplementary material available at 10.1007/s10067-025-07325-y.

**Table:** 

**Key Points** *• Cardiovascular disease risk prediction tools are inaccurate in patients with inflammatory conditions such as rheumatoid arthritis.* *• CVD risk prediction was generally less accurate in people with psoriatic disease than the general population when using QRISK3, Framingham, Raynolds or SCORE tools.* *• QRISK3 tended to achieve the most accurate prediction but was poorly calibrated by overestimating risk.* *• QRISK3 generally overestimated risk; therefore, estimates may not need to be multiplied by 1.5 as recommended for inflammatory arthritis.*			

## Introduction

People living with psoriatic diseases may have increased risk of cardiovascular disease (CVD). Patients with mild psoriasis are associated with increased risk of myocardial infarction and stroke, whilst those with severe psoriasis were additionally associated with risk of cardiovascular mortality [1]. The prevalence of many cardiovascular risk factors is increased in patients with psoriasis, and it is unclear whether psoriasis represents an additional risk for CVD [2]. Patients with psoriatic arthritis (PsA) showed both increased prevalence of many common cardiovascular risk factors, and increased risk of ischemic heart disease, myocardial infarction, stroke and cardiovascular mortality compared to the general population [3]. Effective identification of people at high risk of CVD is essential to encourage lifestyle changes and introduce medications such as statins where required, with the aim of reducing CVD risk.

A number of commonly used CVD risk prediction tools exist including the Framingham risk score (FRS) [4], QRISK3 [5], the Reynolds Risk Score (RRS) [6, 7] and the Systematic Coronary Risk Evaluation score (SCORE) [8]. Current UK guidance advises the assessment of CVD risk every 5 years using QRISK3 [9].

However, there is evidence that risk factors for CVD may differ in patients with inflammatory conditions such as psoriatic disease [10]. Increased inflammation, severity of psoriatic lesions and cytokines involved in plaque formation may be plausible additional risk factors in patients with psoriatic disease [11–13].

It is well established that CVD risk prediction tools often give inaccurate assessment of CVD risk in patients with inflammatory conditions such as rheumatoid arthritis (RA) [14, 15], where QRISK2 often overestimates whilst Framingham, Reynolds and SCORE underestimate CVD risk. The 2017 EULAR recommendations for cardiovascular risk management suggested a multiplier of 1.5 for patients with RA [16]. Similarly, the joint AAD-NPF recommend to use a multiplier of 1.5 for patients with psoriasis with over 10% body surface involvement or who qualify for systemic therapy or polytherapy [17]. Recent work showed some inaccurate CVD prediction in individuals with other inflammatory diseases such as psoriatic disease, although the performance of the SCORE prediction tool was not assessed due to absence of cause of death data [18].

It is crucial to accurately assess CVD risk in order to implement preventative strategies and improve long-term outcomes for patients with psoriatic disease. Hence, assessment of the predictive performance of commonly used CVD risk prediction tools in these cohorts is vital. Our study assesses CVD risk prediction tools in a large cohort of patients with psoriatic disease, including an assessment of the SCORE algorithm for the first time to our knowledge.

## Methods

### Data source

We used data from the UK Biobank, a large prospective cohort study, which recruited just over half a million individuals between 2006 and 2010. This study is part of UK Biobank Project 67547. The UK Biobank study design has been described previously [19]. Briefly, participants were recruited between 2006 and 2010 and aged between 40 and 69. This age range was considered by the Biobank Team to be a compromise between participants being old enough to have experienced disease outcomes, and young enough to prevent diseases from impacting on exposures. Participants attended 22 assessment centres in England, Scotland and Wales where they had blood, urine and saliva samples collected, and answer a touch-screen questionnaire and attended a verbal interview.

### Definitions of inflammatory conditions

In this study, we consider individuals with PsA or psoriasis. Due to recognised concerns that UK Biobank participants may not represent general or disease-specific populations, we also included participants with RA to replicate previous findings of inaccurate CVD risk prediction for RA. We considered patients with none of these conditions as a measure of CVD risk performance in the general population. We excluded from this group any individuals with ankylosing spondylitis since this is similar to both PsA and RA. Individuals were identified as having one of the three inflammatory conditions considered (PsA, psoriasis, RA) if they had a clinical diagnosis using ICD-9 or ICD-10 codes in UK Biobank at or before their baseline visit, or if they self-reported the condition at their initial assessment. Individuals who developed one of the conditions after the baseline visit were excluded.

### Definitions of cardiovascular outcome

Cardiovascular outcomes were determined using ICD-9 or ICD-10 diagnoses in hospital admission data, or through the relevant ICD-10 codes being present as a cause of death. In this study we consider four commonly used CVD risk prediction tools that have previously been assessed in patients with rheumatoid arthritis [14]. The definition of CVD outcome differs slightly for each of QRISK3, FRS, RRS and SCORE, but broadly speaking includes coronary heart disease, cerebro-vascular events, peripheral artery disease and heart failure. Precise definitions can be found in the original model development papers [4–8]. The SCORE tool only predicts fatal CVD events. We defined four CVD outcomes, specific to the definition of each risk prediction tool. Individuals on statins at baseline were removed from the study, as were any individuals who had a record of the relevant CVD event for each risk tool before baseline (the time at which we make a prediction of 10-year risk using the risk prediction tool).

### Risk prediction variables

All risk prediction scores were calculated using the published algorithms for each model. QRISK3 requires the calculation of systolic blood pressure variability. Whilst the UK Biobank study was cross-sectional, two blood pressure measurements were obtained for most individuals at the initial visit (with measurements taken a few moments apart). We estimated variability using these two measurements where possible [20].

The QRISK3 algorithm includes “family history of coronary heart disease in a first degree relative aged less than 60 years old”. The Reynolds risk score uses “parental history of myocardial infarction before age 60”. The UK Biobank lists “heart disease” without further specification; this was taken as the closest approximation to the relevant QRISK3 and RRS predictor variables. A summary of the risk prediction variables required by each model is shown in Table 1.

**Table 1:** Summary of the risk factors included in QRISK3, the Framingham Risk Score, the Reynolds Risk Score and the SCORE CVD risk prediction tools

Risk factor	QRISK3	Framingham Risk Score	Reynolds Risk Score	SCORE
Sex	✓	✓	✓	✓
Age (years)	✓	✓	✓	✓
Body mass index	✓			
Total cholesterol:HDL cholesterol ratio	✓			
Total cholesterol		✓	✓	✓
HDL cholesterol		✓	✓	
Systolic blood pressure	✓	✓	✓	✓
Standard deviation of systolic blood pressure	✓			
Ethnicity	✓			
Smoking status	✓	✓	✓	✓
Family history of coronary heart disease	✓			
Family history of premature myocardial infarction			✓	
Diabetes		✓	✓	
Type 1 diabetes	✓			
Type 2 diabetes	✓			
Treated hypertension	✓	✓		
Rheumatoid arthritis	✓			
Atrial fibrillation	✓			
Chronic kidney disease (stage 3, 4 or 5)	✓			
Migraine	✓			
Corticosteroid use	✓			
Systemic lupus erythematosus	✓			
Atypical antipsychotic use	✓			
Severe mental illness	✓			
Erectile dysfunction or treatments	✓			
Townsend Score	✓			
High sensitivity C-reactive protein			✓	
HbA1c			✓	

### Statistical methods

Data from each individual’s initial Biobank visit were used to calculate each risk prediction score. This is the date at which all the predictor variables are most readily available. An individual’s last observation time was taken to be the earliest of date of Biobank extraction, date of diagnosis of CVD disease or date of death. Follow-up was censored at 10 years to match the prediction window of the risk prediction tools.

Multiple imputation with chained equations (MICE) was used to impute missing data for continuous risk predictors and smoking status. We imputed continuous variables using predictive mean matching, and categorical/binary variables using polytomous/logistic regression. All predictor variables (including interaction terms) for the relevant risk score were used as predictors for the imputations. Five imputed datasets were calculated (to balance computational burden and robustness of results), and results were combined using Rubin’s rules.

We assessed the calibration of the four CVD risk prediction tools by comparing the observed and predicted risks in deciles of predicted risk for each disease group (PsA, psoriasis, RA, or none).

We assessed the ability of each model to distinguish between individuals who experienced CVD events and those who did not using time-dependent area under ROC curve (AUC), sensitivity, specificity, PPV and NPV [21]. These time-dependent approaches account for the fact that not all individuals had 10 years of follow-up data by using inverse probability of censoring weights. We consider a predicted CVD risk of 10% and 20% as thresholds for low-intermediate and intermediate-high CVD risk and report time-dependent sensitivity, specificity, positive predictive value (PPV) and negative predictive value (NPV) at both thresholds [14]. All analysis was conducted in R (version 3.6.2).

## Results

**Fig. 1:**
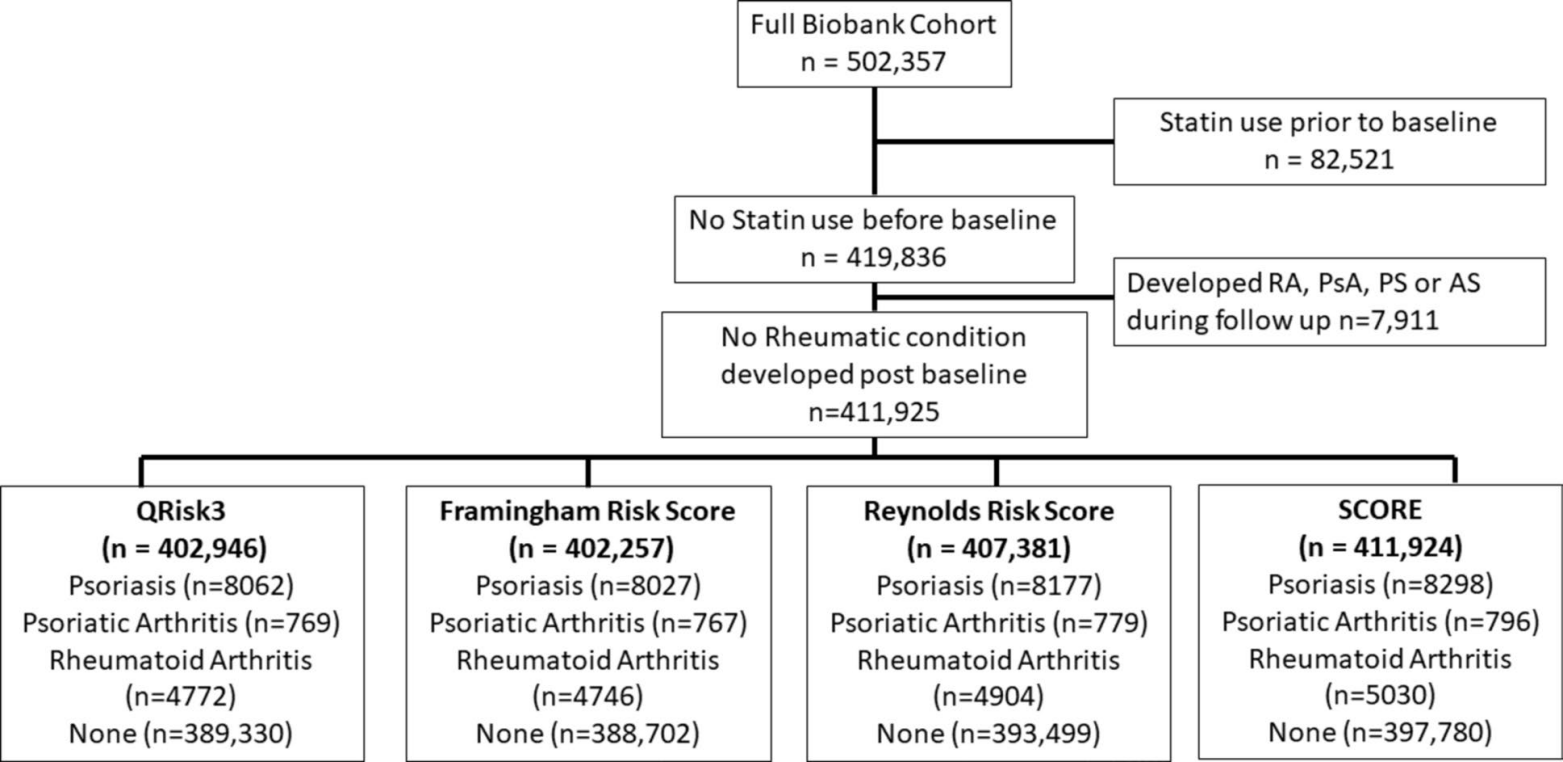
Flow chart showing inclusion/exclusion of individuals for the analysis cohort for each risk tool

The Biobank cohort consisted of 502,357 individuals. We removed 82,521 individuals who were prescribed statins before their initial Biobank visit and 7911 individuals who developed an inflammatory condition during follow-up. Individuals with evidence of a CVD condition prior to their Biobank visit or with missing Townsend Score for the QRISK3 cohort were removed, as was one individual with missing sex. Full details of the cohorts under consideration for each risk tool are shown in Fig. 1, with individual characteristics described in Supplementary Table S1.

### CVD incidence

**Fig. 2:**
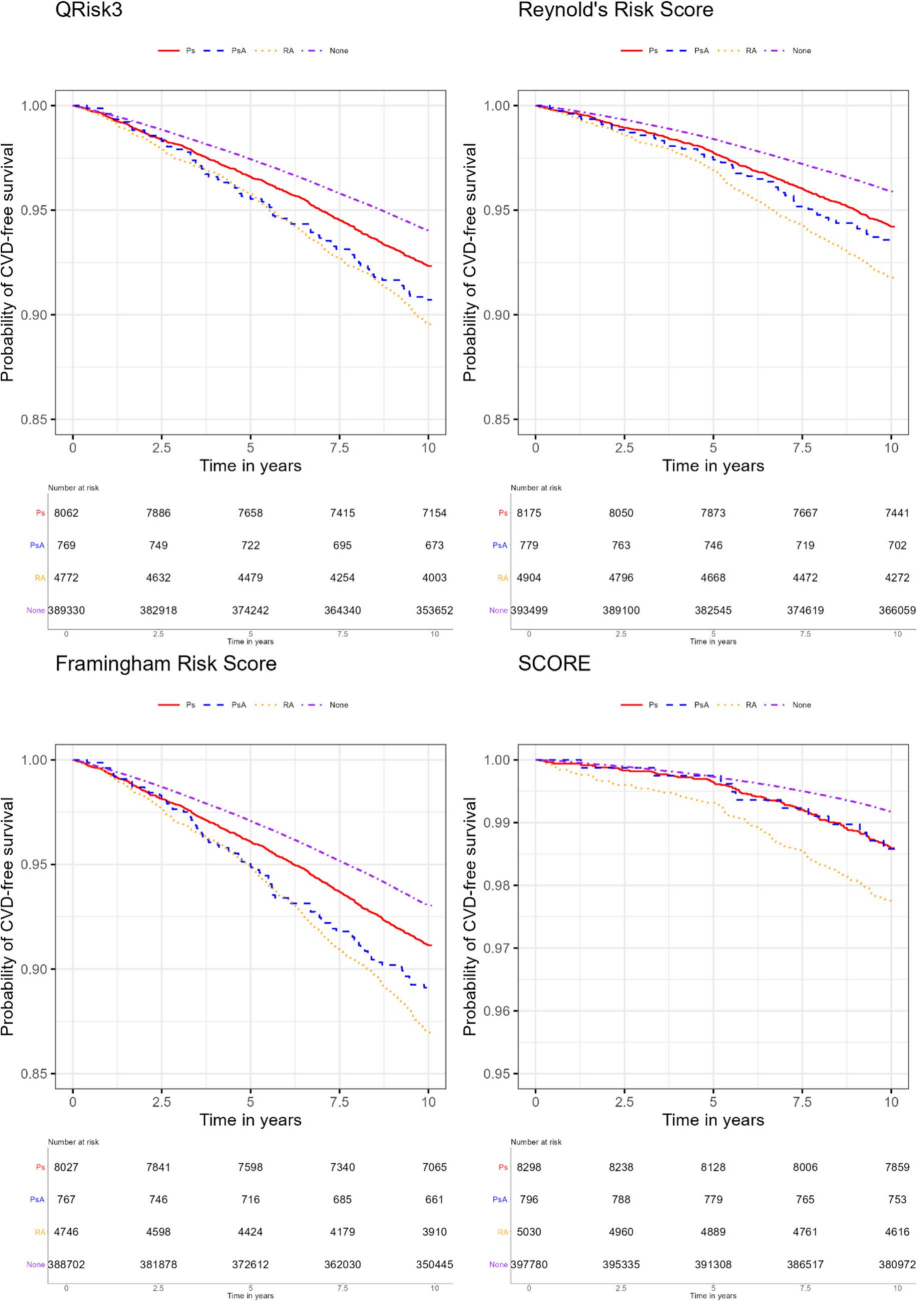
The Kaplan–Meier curves showing incidence of CVD events for each disease according to the four risk tool definitions

The Kaplan–Meier curves showing the incidence of CVD events during follow-up following the initial Biobank visit for each cohort are shown in Fig. 2. CVD incidence was increased for individuals with psoriasis, PsA or RA, irrespective of which specific definition was used, compared to individuals with none of the inflammatory conditions. Interestingly, according to the QRISK3 and Framingham risk score definitions of CVD events, individuals with PsA were more similar to individuals with RA in terms of observed CVD incidence. However, for the Reynolds risk score and SCORE definitions, individuals with PsA had CVD incidence which was more similar to individuals with psoriasis.

Each of the inflammatory conditions resulted in generally slightly higher risk predictions than those obtained for individuals with none of the inflammatory conditions, with individuals with RA usually allocated the highest risk scores in general. Boxplots of the calculated risk scores for each risk tool according to disease definition is shown in Supplementary Fig. S1.

### Risk discrimination

**Fig. 3:**
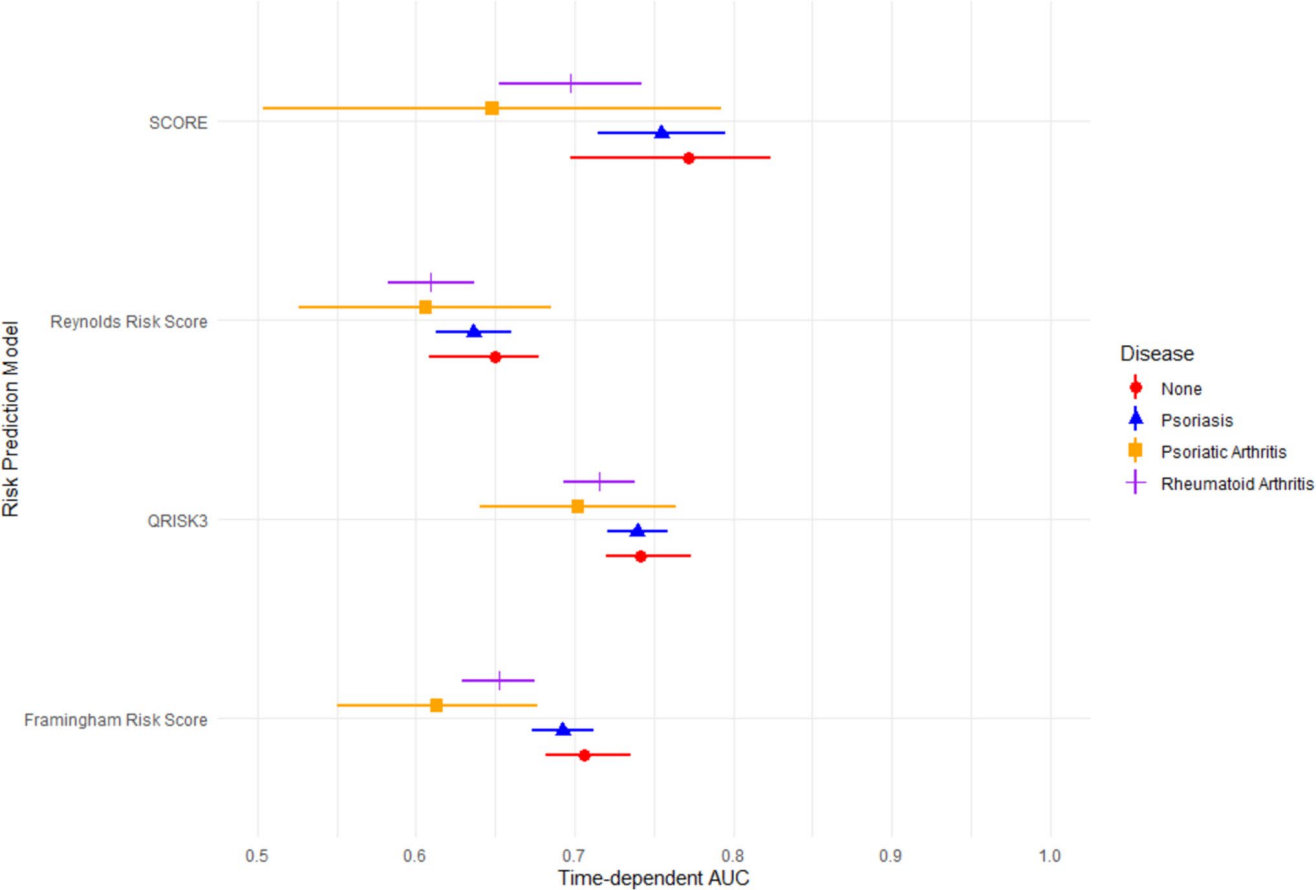
Time-dependent area under ROC curve (AUC) for risk prediction tool and disease cohort. Point estimates and 95% confidence intervals are shown

We assessed the discrimination of each CVD risk prediction tool using time-dependent AUC. Each risk prediction tool was most accurate for individuals with none of the three inflammatory conditions under consideration, although performance in participants with psoriasis was generally similar (Fig. 3). ROC curves, showing time-dependent sensitivity and specificity, are shown in Supplementary Fig. S2.

For example, for the QRISK3 tool, the group with no inflammatory disease achieved a time-dependent AUC of 0.74 (Table 2, 95% confidence interval (0.720, 0.774)). In contrast, individuals with PsA obtained the least accurate predictions with an AUC of 0.70 (95%CI, 0.640, 0.764). Individuals with RA also obtained less accurate predictions with AUC of 0.72 (0.693, 0.738). Individuals with psoriasis obtained predictions that were roughly as accurate as those with no inflammatory conditions with AUC of 0.74 (95%CI 0.720, 0.759). Broadly similar patterns are observed for each of the four CVD risk prediction tools.

**Table 2:** Prediction accuracy of each CVD risk tool at 10% and 20% thresholds, for each disease group

		10% risk threshold					20% risk threshold			
Risk Score	Disease	AUC	Sensitivity	Specificity	PPV	NPV	Sensitivity	Specificity	PPV	NPV
QRISK3	PSA	0.702	0.674	0.574	0.139	0.945	0.320	0.887	0.225	0.927
	Ps	0.740	0.735	0.591	0.130	0.964	0.368	0.881	0.204	0.944
	RA	0.716	0.846	0.451	0.152	0.962	0.465	0.797	0.211	0.927
	None	0.741	0.707	0.649	0.113	0.972	0.304	0.911	0.179	0.954
Framingham Risk Score	PSA	0.613	0.780	0.313	0.122	0.921	0.439	0.707	0.155	0.912
	Ps	0.692	0.884	0.335	0.114	0.967	0.570	0.688	0.151	0.943
	RA	0.652	0.840	0.343	0.161	0.934	0.479	0.724	0.206	0.903
	None	0.706	0.868	0.384	0.095	0.975	0.549	0.734	0.134	0.956
Reynolds Risk Score	PSA	0.606	0.103	0.932	0.094	0.938	0.041	0.980	0.125	0.937
	Ps	0.636	0.111	0.942	0.105	0.945	0.045	0.989	0.200	0.944
	RA	0.609	0.191	0.881	0.126	0.924	0.047	0.978	0.159	0.920
	None	0.650	0.103	0.949	0.080	0.961	0.025	0.991	0.111	0.960
SCORE	PSA	0.648	0.092	0.982	0.068	0.987	0.000	1.000	0.000	0.986
	Ps	0.755	0.158	0.970	0.071	0.988	0.021	0.998	0.126	0.986
	RA	0.697	0.075	0.971	0.056	0.978	0.013	0.997	0.076	0.978
	None	0.772	0.143	0.974	0.044	0.993	0.020	0.998	0.073	0.992

Individuals with psoriatic arthritis or rheumatoid arthritis consistently achieved lower AUC values than those with no inflammatory disease. However, the confidence intervals overlapped for all tools except the Framingham risk score, where individuals with PsA or RA had significantly lower AUC than individuals with no inflammatory disease (Fig. 3). In terms of discrimination, individuals with psoriasis achieved predictions of roughly comparable accuracy to the general population. Large confidence intervals for the cohort with PsA, likely due to the small sample size of this cohort, prevent similar conclusions being drawn for the other risk tools.

### Risk calibration

**Fig. 4:**
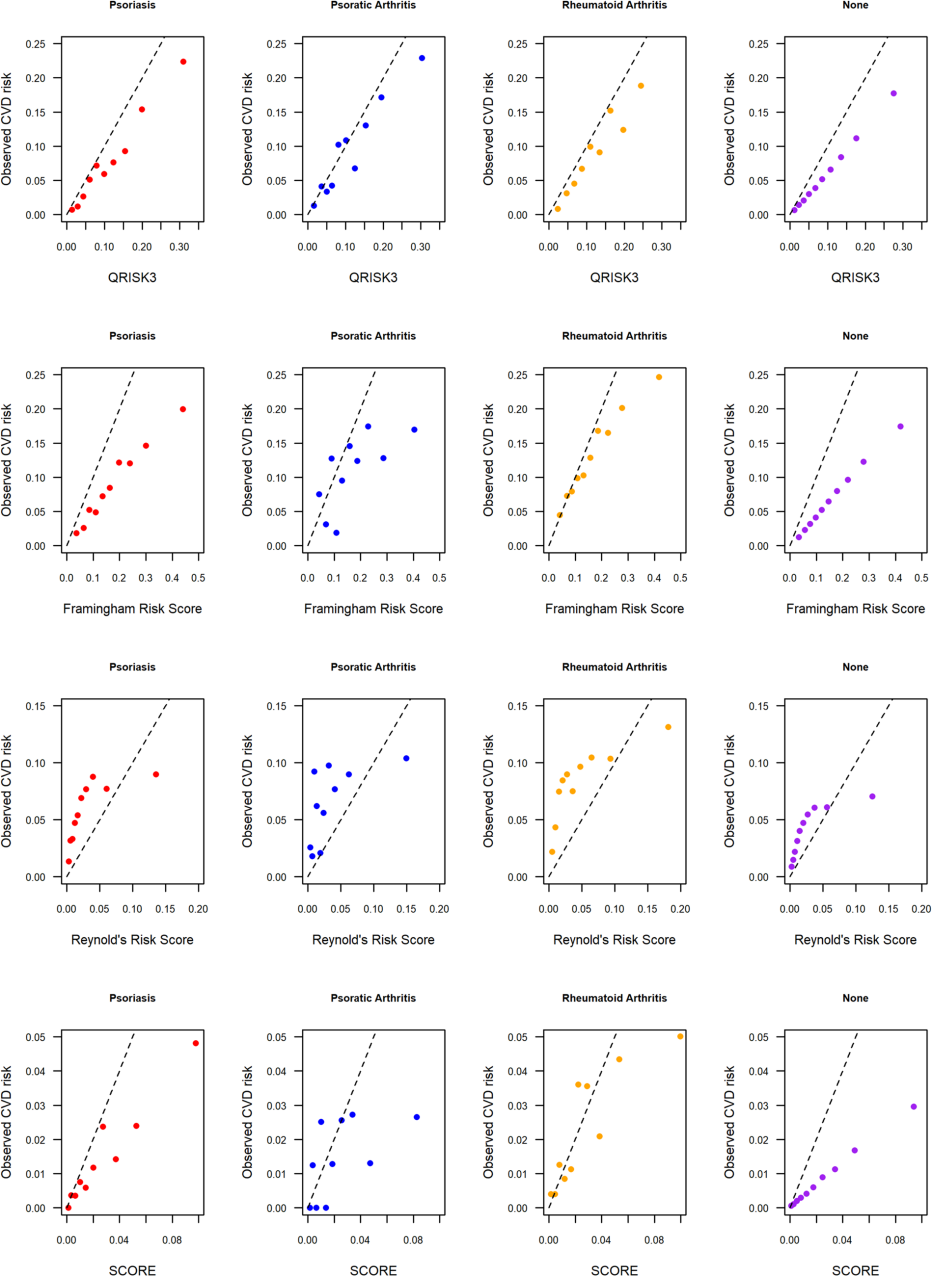
Calibration plots comparing observed CVD risk in deciles of predicted CVD risk using each of the four CVD risk tools, and for each disease cohort

We compared the observed and predicted CVD risk to assess calibration of each CVD risk prediction tool (Fig. 4). Overall, the predictions for the UK Biobank cohort were not well calibrated, with the least calibrated predictions occurring in the group with no inflammatory disease. QRISK3 and Framingham risk score tended to overpredict CVD risk in each decile of predicted risk. The Reynolds risk score tended to underpredict CVD risk. Results were mixed for the SCORE risk prediction tool. Predictions for participants with psoriasis or no inflammatory condition were generally overpredicted. No clear pattern was observed for individuals with PsA or RA for whom there was a mix of over and under prediction across the deciles of predicted risk. Interestingly, results were most overpredicted for those with no inflammatory conditions. CVD risk predictions using QRISK3 were most overestimated for the highest risk individuals with psoriasis and RA, but reasonably well calibrated for lower risk individuals.

## Discussion

We assessed the predictive accuracy of four common CVD risk prediction tools in UK Biobank participants with psoriatic arthritis or psoriasis. To contextualise these findings, we also replicated previous findings concerning less accurate predictions for rheumatoid arthritis, and compared the performance in patients with none of these conditions. The first finding of note was that none of the CVD risk prediction tools validated well in the UK Biobank cohort. This agrees with a recent study showing that QRISK3 achieves moderate discrimination (ability to distinguish CV events from non-events) in the UK Biobank, and extends this finding to the Framingham Risk Score, Reynolds Risk Score and SCORE [22]. We also showed that predictions of CVD risk were less accurate (in terms of time-dependent AUC) for individuals with psoriasis, PsA or RA than they are for the general population. Due to the small sample size for PsA in particular, only for the Framingham risk score were the confidence intervals for time-dependent AUCs non-overlapping between individuals with PsA (or RA) and individuals with none of the inflammatory diseases under consideration. However, the pattern of reduced discriminative ability for PsA and RA was consistent across tools. QRISK3 tended to achieve the most accurate AUCs for each disease cohort, but was poorly calibrated, often overpredicting CVD risk.

The UK Biobank is known to be less representative of the general population. Patients are generally older, healthier and less socioeconomically deprived than the average in the UK [23].

The original QRISK3 paper reported C-index of 0.880 for women and 0.858 for men [5]. The Framingham Study reported C-index of 0.763 for men and 0.793 for women [4]. The C-index is conceptually similar to the time-dependent AUC we use in this study, although it does not account for censoring caused by not having 10 years of observation on all patients to determined CVD status. It also ignores pairs of observations where the individual with shorter observation period did not experience the event [24]. The Reynolds risk score did not publish AUC data for their model, whilst the SCORE algorithm reported an AUC of between 0.71 and 0.84 for various training and validation cohorts [8]. None of the cohorts in our study achieved discriminative accuracy comparable to the performance reported in the original models.

In addition to poor discriminative ability, we observed poor calibration in our analysis. QRISK3, SCORE and the Framingham risk score generally overestimated risks whilst the Reynolds Risk score under predicted risk. Poorly calibrated models do not provide a true assessment of an individual’s risk which may have negative effects if preventative decisions are made based on risk assessments.

Our observation of poorly calibrated risk predictions has also been observed previously for RA patients. Arts et al. [14] report underestimated risks for Framingham Risk Score, Reynolds Risk Score and SCORE, and overestimated risks for QRISK2 for individuals with RA. We obtained similar results for QRISK3 and Reynolds risk score, but in contrast found generally overestimated risks for Framingham Risk Score and a more mixed picture for SCORE [14]. This may be because the predicted risks obtained in our Biobank cohort were generally lower than those obtained in the Nijmegen early rheumatoid arthritis inception cohort.

The findings of our study that QRISK3 overpredicts CVD risk and the Reynolds risk score underpredicts in patients with psoriatic arthritis are in agreement with an Italian study of 155 individuals with psoriatic arthritis [12]. However, the accuracy of predictions was worse in our study than in their cohort. They also suggest that SCORE and Framingham Risk Score underpredict CVD risk, which contrasts with our observations in the UK Biobank.

The findings of this study broadly agree with a recent validation of CVD risk prediction tools in patients with rheumatic and psoriatic disease using primary care data from the Clinical Practice Research Datalink (CPRD). Our study adds to this work by also considering the SCORE risk prediction tool (due to the availability of cause of death data), and through the comparison of prediction performance to individuals with no psoriatic/rheumatic disease [18].

Our study suggests that the UK Biobank often systematically overpredicts CVD risk, across the different disease cohorts. This may mean that UK Biobank is of less use to validated risk prediction models since the participants are likely to be more healthy than the individuals who would usually be assessed using CVD risk prediction tools [25].

Each CVD risk prediction tool uses different information about a patient’s risk factors (Table 1), with QRISK3 using many more risk factors than the other tools. In addition, even where the same risk factors are included in a model, the weightings attached to them in each model can be very different. This may account for some of the differences between the various tools. The inclusion of more risk factors in QRISK3 may be one feature that leads to over prediction of risk.

A limitation of the UK Biobank data is the fact that some of the outcome data is self-reported. This may limit the accuracy of diagnoses, both of the CVD outcomes, the inflammatory conditions, and the presence or absence of risk factors. Nevertheless, the fact that our findings are broadly in line with other studies in this area suggests that this limitation has not overly affected the conclusions drawn.

CVD risk is an important part of psoriatic disease management. Our results have shown that existing risk prediction tools have limitations in giving accurate assessments of an individual’s risk. This may have implications for primary prevention, and overtreatment for example with tools that over predict risk. For example, prescription of statins to individuals with greater than 10% risk may lead to over-prescription in patients with psoriatic disease. By contrast, under prediction of risk would be problematic, as it would lead to missing patients that would benefit from preventative measures. There may be a need for disease-specific risk prediction tools. However, the risk factors responsible for increased CVD risk would need to be identified, and will likely differ for PsA, psoriasis and RA [10]. Initial efforts to develop disease-specific CVD risk prediction tools for rheumatoid arthritis have proved challenging [26–29]. Inflammatory markers and disease activity may be useful additional risk factors [30]. Recent work combining the results of different prediction models offers promising results [31, 32]. Further work may be needed in this area. However, in the meantime, clinicians should be aware of the overprediction of CVD risk in patients with psoriatic disease using the QRISK3 tool.

## Supplementary Information

Below is the link to the electronic supplementary material.

Supplementary File1 (DOCX 282 KB)

## Data Availability

This research has been conducted using the UK Biobank Resource under application number 67547. Requests to access the data should be made directly to the UK Biobank.
