# Supplementary material for: External validation of the accuracy of cardiovascular risk prediction tools in psoriatic disease: a UK Biobank study
Source: Clin Rheumatol. 2025 Jan 20;44(3):1151–61. doi: 10.1007/s10067-025-07325-y (PMC11865138; doi:10.1007/s10067-025-07325-y)
Supplement: Supplementary file 1 — Supplementary File1 (DOCX 282 KB) [file 10067_2025_7325_MOESM1_ESM.docx]

**Supplement to: External validation of the accuracy of cardiovascular risk prediction tools in psoriatic disease: A UK Biobank Study**


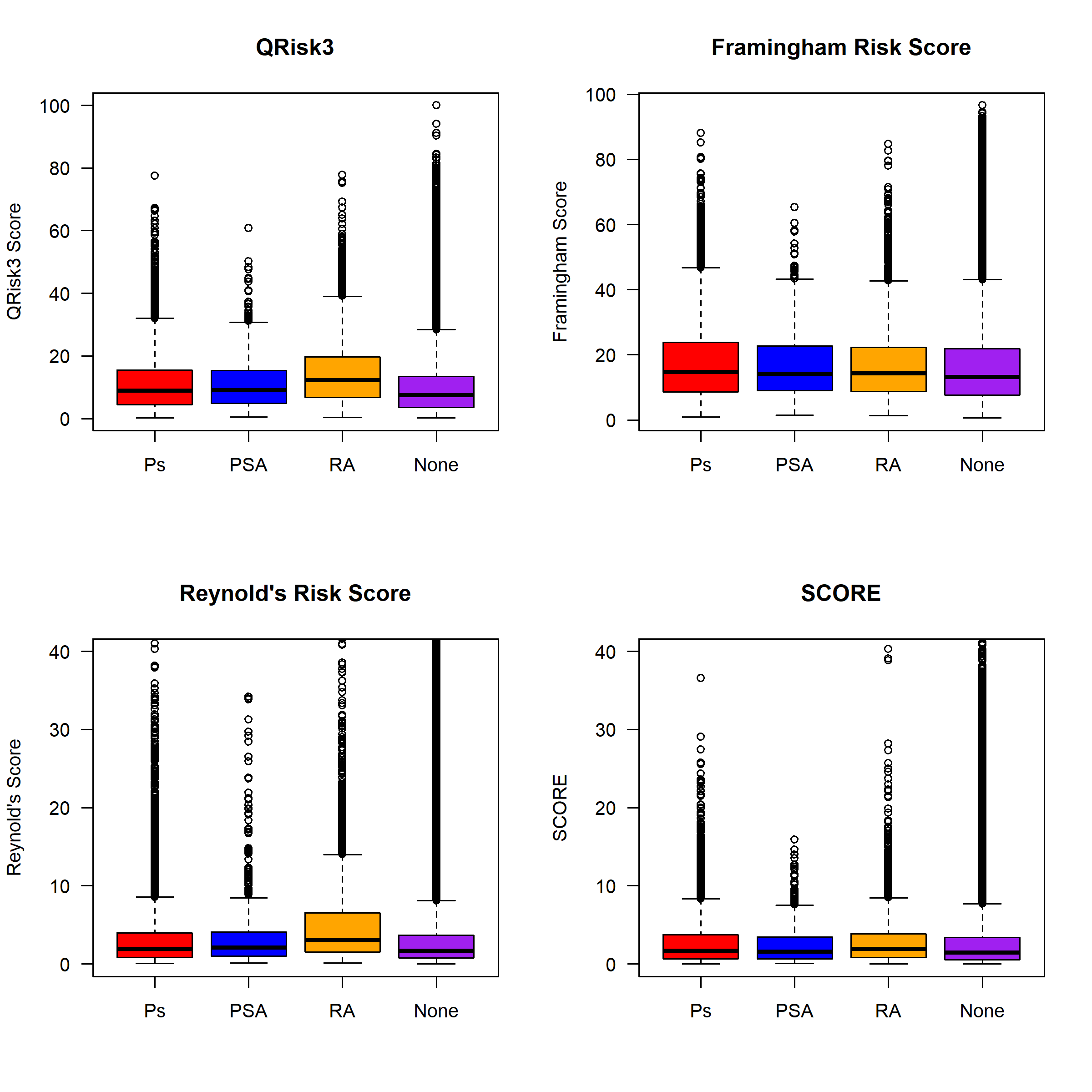


Figure S1:Boxplots showing predicted risk by disease category for each of the CVD risk tools. The results shown are from one of the 5 imputed datasets, with the results for the remaining four imputed datasets showing similar trends.


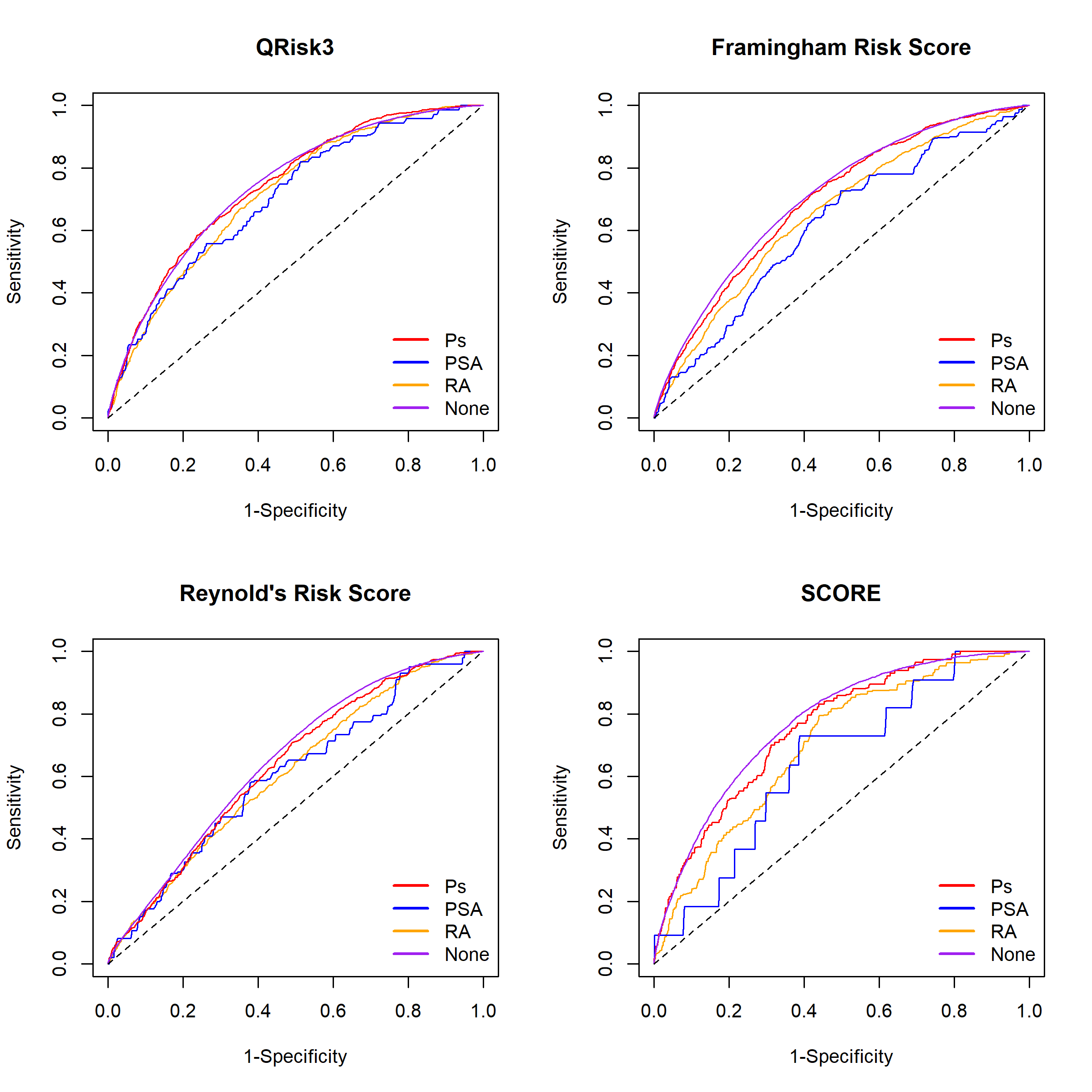


Figure S2: ROC curves for each disease definition for each CVD Risk prediction tool.

Table S1: Characteristics of the UK Biobank cohort for each CVD risk tool

|  | QRisk3 CVD Disease | | | Framingham CVD Risk | | | Reynold’s Risk Score CVD Risk | | | SCORE CVD Risk | | |
| --- | --- | --- | --- | --- | --- | --- | --- | --- | --- | --- | --- | --- |
| Risk Factor | No | Yes | p | No | Yes | p | No | Yes | p | No | Yes | p |
| Total N (%) | 378927 (94.0) | 24019 (6.0) | <0.001 | 374238 (93.0) | 28019 (7.0) | <0.001 | 390575 (95.9) | 16806 (4.1) | <0.001 | 408467 (99.2) | 3457 (0.8) | <0.001 |
| Psoriasis | 7455 (2.0) | 607 (2.5) | <0.001 | 7327 (2.0) | 700 (2.5) | <0.001 | 7710 (2.0) | 465 (2.8) | <0.001 | 8184 (2.0) | 114 (3.3) | <0.001 |
| Psoriatic Arthritis | 699 (0.2) | 70 (0.3) | <0.001 | 685 (0.2) | 82 (0.3) | <0.001 | 730 (0.2) | 49 (0.3) | 0.003 | 785 (0.2) | 11 (0.3) | 0.137 |
| Rheumatoid Arthritis | 4287 (1.1) | 485 (2.0) | <0.001 | 4142 (1.1) | 604 (2.2) | <0.001 | 4510 (1.2) | 394 (2.3) | <0.001 | 4920 (1.2) | 110 (3.2) | <0.001 |
| Female | 222101 (58.6) | 9562 (39.8) | <0.001 | 219783 (58.7) | 11577 (41.3) | <0.001 | 228111 (58.4) | 5994 (35.7) | <0.001 | 234849 (57.5) | 1080 (31.2) | <0.001 |
| Age | 55.2 (8.1) | 59.7 (7.0) | <0.001 | 55.1 (8.1) | 59.8 (7.0) | <0.001 | 55.3 (8.1) | 60.1 (7.0) | <0.001 | 55.5 (8.1) | 61.6 (6.5) | <0.001 |
| **Ethnicity**  White/not recorded | 358956 (94.7) | 22842 (95.1) | <0.001 | 354444 (94.7) | 26675 (95.2) | <0.001 | 369844 (94.7) | 16034 (95.4) | <0.001 | 386865 (94.7) | 3299 (95.4) | 0.017 |
| Other | 5973 (1.6) | 302 (1.3) |  | 5916 (1.6) | 357 (1.3) |  | 6175 (1.6) | 187 (1.1) |  | 6374 (1.6) | 45 (1.3) |  |
| Other Asian | 1248 (0.3) | 95 (0.4) |  | 1241 (0.3) | 103 (0.4) |  | 1303 (0.3) | 59 (0.4) |  | 1366 (0.3) | 8 (0.2) |  |
| Chinese | 1348 (0.4) | 27 (0.1) |  | 1341 (0.4) | 38 (0.1) |  | 1366 (0.3) | 21 (0.1) |  | 1386 (0.3) | 8 (0.2) |  |
| Indian | 3891 (1.0) | 349 (1.5) |  | 3853 (1.0) | 379 (1.4) |  | 4090 (1.0) | 231 (1.4) |  | 4336 (1.1) | 44 (1.3) |  |
| Pakistani | 1162 (0.3) | 125 (0.5) |  | 1149 (0.3) | 135 (0.5) |  | 1244 (0.3) | 73 (0.4) |  | 1336 (0.3) | 16 (0.5) |  |
| Bangladeshi | 136 (0.0) | 18 (0.1) |  | 134 (0.0) | 19 (0.1) |  | 143 (0.0) | 15 (0.1) |  | 160 (0.0) | 0 (0.0) |  |
| Black Caribbean | 3537 (0.9) | 159 (0.7) |  | 3498 (0.9) | 189 (0.7) |  | 3641 (0.9) | 111 (0.7) |  | 3778 (0.9) | 28 (0.8) |  |
| Black African | 2676 (0.7) | 102 (0.4) |  | 2662 (0.7) | 124 (0.4) |  | 2769 (0.7) | 75 (0.4) |  | 2866 (0.7) | 9 (0.3) |  |
| **Smoking Status**  former smoker | 180797 (47.7) | 11750 (48.9) | <0.001 | 178528 (47.7) | 13655 (48.7) | <0.001 | 186657 (47.8) | 8079 (48.1) | <0.001 | 195375 (47.8) | 1660 (48.0) | <0.001 |
| heavy smoker | 8830 (2.3) | 1206 (5.0) |  | 8527 (2.3) | 1457 (5.2) |  | 9178 (2.3) | 1031 (6.1) |  | 10164 (2.5) | 300 (8.7) |  |
| light smoker | 17924 (4.7) | 1316 (5.5) |  | 17666 (4.7) | 1544 (5.5) |  | 18381 (4.7) | 1057 (6.3) |  | 19407 (4.8) | 272 (7.9) |  |
| moderate smoker | 10878 (2.9) | 1088 (4.5) |  | 10606 (2.8) | 1319 (4.7) |  | 11218 (2.9) | 886 (5.3) |  | 12109 (3.0) | 216 (6.2) |  |
| non-smoker | 158554 (41.8) | 8477 (35.3) |  | 157011 (42.0) | 9824 (35.1) |  | 163103 (41.8) | 5625 (33.5) |  | 169228 (41.4) | 973 (28.1) |  |
| (Missing) | 1944 (0.5) | 182 (0.8) |  | 1900 (0.5) | 220 (0.8) |  | 2038 (0.5) | 128 (0.8) |  | 2184 (0.5) | 36 (1.0) |  |
| Atrial Fibrillation | 2890 (0.8) | 744 (3.1) | <0.001 | 2507 (0.7) | 919 (3.3) | <0.001 | 3351 (0.9) | 608 (3.6) | <0.001 | 4123 (1.0) | 260 (7.5) | <0.001 |
| Antipsychotic medication use | 957 (0.3) | 88 (0.4) | 0.001 | 932 (0.2) | 112 (0.4) | <0.001 | 989 (0.3) | 70 (0.4) | <0.001 | 1056 (0.3) | 32 (0.9) | <0.001 |
| Corticosteroid use | 3805 (1.0) | 456 (1.9) | <0.001 | 3675 (1.0) | 567 (2.0) | <0.001 | 3971 (1.0) | 401 (2.4) | <0.001 | 4389 (1.1) | 111 (3.2) | <0.001 |
| Erectile Dysfunction | 1163 (0.3) | 152 (0.6) | <0.001 | 1136 (0.3) | 171 (0.6) | <0.001 | 1228 (0.3) | 122 (0.7) | <0.001 | 1361 (0.3) | 39 (1.1) | <0.001 |
| Migraine | 16240 (4.3) | 948 (3.9) | 0.012 | 16070 (4.3) | 1082 (3.9) | 0.001 | 16856 (4.3) | 570 (3.4) | <0.001 | 17595 (4.3) | 83 (2.4) | <0.001 |
| Chronic Kidney Disease Stage 3,4 or 5 | 9257 (2.4) | 1000 (4.2) | <0.001 | 8973 (2.4) | 1195 (4.3) | <0.001 | 9657 (2.5) | 813 (4.8) | <0.001 | 10536 (2.6) | 310 (9.0) | <0.001 |
| Severe Mental Illness | 749 (0.2) | 75 (0.3) | <0.001 | 733 (0.2) | 93 (0.3) | <0.001 | 774 (0.2) | 64 (0.4) | <0.001 | 832 (0.2) | 35 (1.0) | <0.001 |
| Systemic lupus erythematosus | 539 (0.1) | 66 (0.3) | <0.001 | 530 (0.1) | 73 (0.3) | <0.001 | 577 (0.1) | 46 (0.3) | <0.001 | 633 (0.2) | 12 (0.3) | 0.009 |
| Anti-hypertensive Medication | 46424 (12.3) | 5873 (24.5) | <0.001 | 44976 (12.0) | 6909 (24.7) | <0.001 | 49689 (12.7) | 4329 (25.8) | <0.001 | 54831 (13.4) | 1262 (36.5) | <0.001 |
| Diabetes | 6249 (1.6) | 1023 (4.3) | <0.001 | 6030 (1.6) | 1185 (4.2) | <0.001 | 6784 (1.7) | 883 (5.3) | <0.001 | 7857 (1.9) | 351 (10.2) | <0.001 |
| Type-1 Diabetes | 750 (0.2) | 197 (0.8) | <0.001 | 718 (0.2) | 223 (0.8) | <0.001 | 857 (0.2) | 169 (1.0) | <0.001 | 1055 (0.3) | 91 (2.6) | <0.001 |
| Type-2 Diabetes | 5499 (1.5) | 826 (3.4) | <0.001 | 5312 (1.4) | 962 (3.4) | <0.001 | 5927 (1.5) | 714 (4.2) | <0.001 | 6802 (1.7) | 260 (7.5) | <0.001 |
| Weight (kg) | 76.5 (15.4) | 80.8 (16.0) | <0.001 | 76.5 (15.4) | 80.8 (16.4) | <0.001 | 76.6 (15.4) | 81.3 (16.6) | <0.001 | 76.8 (15.5) | 82.7 (18.9) | <0.001 |
| Height (cm) | 168.3 (9.3) | 169.6 (9.4) | <0.001 | 168.3 (9.3) | 169.4 (9.4) | <0.001 | 168.3 (9.3) | 170.0 (9.3) | <0.001 | 168.4 (9.3) | 170.2 (9.5) | <0.001 |
| Family History of Heart Disease | 150033 (39.6) | 11645 (48.5) | <0.001 | 148014 (39.6) | 13315 (47.5) | <0.001 | 156279 (40.0) | 7797 (46.4) | <0.001 | 164837 (40.4) | 1486 (43.0) | 0.002 |
| Systolic Blood Pressure (SBP, mm HG) | 138.3 (19.5) | 146.7 (20.4) | <0.001 | 138.2 (19.5) | 146.5 (20.5) | <0.001 | 138.5 (19.6) | 147.3 (20.7) | <0.001 | 138.8 (19.7) | 148.3 (22.0) | <0.001 |
| SBP variability | 5.3 (4.4) | 5.8 (4.7) | <0.001 | 5.3 (4.4) | 5.8 (4.7) | <0.001 | 5.3 (4.4) | 5.8 (4.8) | <0.001 | 5.3 (4.4) | 6.0 (5.0) | <0.001 |
| Townsend Deprivation Score | -1.4 (3.0) | -1.2 (3.2) | <0.001 | -1.4 (3.0) | -1.1 (3.2) | <0.001 | -1.4 (3.0) | -1.1 (3.2) | <0.001 | -1.4 (3.0) | -0.4 (3.5) | <0.001 |
| Total Cholesterol (mmol/L) | 5.9 (1.1) | 6.1 (1.1) | <0.001 | 5.9 (1.1) | 6.0 (1.1) | <0.001 | 5.9 (1.1) | 6.0 (1.1) | <0.001 | 5.9 (1.1) | 5.8 (1.2) | <0.001 |
| HDL Cholesterol (mmol/L) | 1.5 (0.4) | 1.4 (0.4) | <0.001 | 1.5 (0.4) | 1.4 (0.4) | <0.001 | 1.5 (0.4) | 1.4 (0.4) | <0.001 | 1.5 (0.4) | 1.4 (0.4) | <0.001 |
| HbA1c (%) | 5.4 (0.5) | 5.5 (0.6) | <0.001 | 5.4 (0.5) | 5.5 (0.6) | <0.001 | 5.4 (0.5) | 5.5 (0.7) | <0.001 | 5.4 (0.5) | 5.7 (0.9) | <0.001 |
| CRP (mg/L) | 2.5 (4.2) | 3.3 (5.0) | <0.001 | 2.4 (4.1) | 3.4 (5.2) | <0.001 | 2.5 (4.2) | 3.6 (5.6) | <0.001 | 2.5 (4.2) | 4.5 (6.8) | <0.001 |
